# The activity view of inner speech

**DOI:** 10.3389/fpsyg.2015.00232

**Published:** 2015-03-09

**Authors:** Fernando Martínez-Manrique, Agustín Vicente

**Affiliations:** ^1^Departamento de Filosofía I, University of Granada, Granada, Spain; ^2^Ikerbasque: Basque Foundation for Science, Bilbao, Spain; ^3^Department of Linguistics and Basque Studies, University of the Basque Country, Vitoria, Spain

**Keywords:** inner speech, format view, activity view, consciousness, unsymbolized thinking, phonological representation, action prediction

## Abstract

We distinguish two general approaches to inner speech (IS)—the “format” and the “activity” views—and defend the activity view. The format view grounds the utility of IS on features of the representational format of language, and is related to the thesis that the proper function of IS is to make conscious thinking possible. IS appears typically as a product constituted by representations of phonological features. The view also has implications for the idea that passivity phenomena in cognition may be misattributed IS. The activity view sees IS as a speaking activity that does not have a proper function in cognition. It simply inherits the array of functions of outer speech. We argue that it is methodologically advisable to start from this variety of uses, which suggests commonalities between internal and external activities. The format view has several problems; it has to deny “unsymbolized thinking”; it cannot easily explain how IS makes thoughts available to consciousness, and it cannot explain those uses of IS where its format features apparently play no role. The activity view not only lacks these problems but also has explanatory advantages: construing IS as an activity allows it to be integrally constituted by its content; the view is able to construe unsymbolized thinking as part of a continuum of phenomena that exploit the same mechanisms, and it offers a simple explanation for the variety of uses of IS.

## INTRODUCTION

Inner speech (IS) is typically characterized as the experience of silently talking to oneself. It is reported as phenomenologically different from other experiences such as visual images, emotions, or the controversial phenomenon of unsymbolized thought (Hurlburt and Akhter, 2008). In this paper we distinguish two general approaches to IS—what we will call the “format” and the “activity” views. These approaches hold different theses about what elements are more relevant to characterize the phenomenon. As we will see, the format view regards IS chiefly as a certain product with certain format features, whereas the activity view emphasizes its properties as an activity. These may appear as mere differences in emphasis—after all, the format view may readily accept that IS is an activity and the activity view does not deny that there is a format involved. Yet the reason for their respective emphases lies in the fact that they have distinct commitments to what is central of the phenomenon. In particular, we will see that the two approaches have different views concerning the cognitive functions of IS, especially whether IS is or is not necessary for conscious thinking.

These are, in general, philosophical approaches, yet empirically well-informed ones. We are aware that, on the one hand, as a verbal phenomenon, a good account of IS will ultimately depend on precise models of linguistic production and comprehension; and that, on the other hand, as a cognitive phenomenon, a plausible account of IS requires more data than we presently have. However, it is useful to bring to the light the commitments and consequences of holding a certain general view of what IS actually is. In particular, it helps for the methodological assessment of what aspects of the phenomenon it is worthwhile to investigate. In this paper we spell out the differences between the format and activity views, and defend the advantages of the latter.

## THE FORMAT VIEW OF INNER SPEECH

The format view is attributable to most authors who have written about the functions of IS in the last two decades^1^. In its strongest form,^2^ it can be characterized by the following three theses:

(i) the strong consciousness thesis: IS is necessary for conscious thinking;
(ii) the format thesis: in IS we recruit a representational system because of its features as a format;
(iii) the product thesis: IS consists in some output of the linguistic production system, typically strings of phonological representations.

The first thesis is about the *role* of IS. If “thinking” is roughly understood as any cognitive event that involves the manipulation or tokening of propositional contents, the thesis says that doing any of this consciously requires the presence of IS. The second thesis is about the *nature* of IS. It says that what is essential for something to count as IS is that it is formatted in a certain way. The third thesis provides a further specification of the kinds of representations involved in IS.

The first and second theses are two sides of the same coin: it is claimed that in IS we recruit a format with certain features because those features open the possibility to have conscious thoughts^3^ at all. Different authors have focused on different features, such as digitality, or context-independence (Clark, 1998), perceptuality and introspectability (Jackendoff, 1996, 2012; Prinz, 2011, 2012; Bermúdez, 2003), and predicative structure (Bermúdez, 2003). To take one example: Jackendoff, and Prinz following him, holds that “pure” conscious thinking is impossible for architectural reasons: we can be conscious of intermediate level representations (like 2.5D representations in the visual system), but never of basic-level or higher-level representations, such as concepts or spatial 3D representations. Thus, if we want to have conscious thoughts, we have to use a representational format that has the right kind of representations. Images are good, but phonological representations are much better, given that phonological representations can vehicle many more kinds of thoughts (about the future or past, about *abstracta* and *possibilia*, about relations, etc.).

These considerations lead Jackendoff to the product thesis, i.e., that IS is constituted by strings of phonological representations or structures^4^. One may wonder, however, how central the product thesis is for the format view, and how specific its commitment to a certain type of product is. With respect to centrality, one may contend that the view does not need to regard IS as constituted solely by phonological representations^5^. Surely IS appears as a content-carrying format so it is also constituted by a semantic component. Moreover, the general approach can also be formulated in a way that is compatible with the idea that IS is an action: the action of producing strings of inner linguistic items (mainly) with the purpose of bringing our thoughts to consciousness. In fact, sometimes Carruthers (2011) comes close to presenting IS in this way, so depicting him as endorsing the format view can seem contentious. The difference between this view and what we will call the activity view would perhaps appear as a matter of emphasis and degree.

However, Carruthers (2014), as Jackendoff, Prinz, or Bermúdez, does put the focus on the product and its properties^6^. It has to be noted, on the other hand, that many authors who are not particularly concerned with the issue of the role of IS in conscious thinking, also take IS to be a product (Pickering and Garrod, 2013). That is, it seems to be customary to think of IS as a product and not as an activity of some kind. With respect to the commitment to a specific kind of product, one may observe that there are different kinds of phonological representations. We can distinguish at least articulatory, phonemic, and acoustic phonological representations. We may think that the activity of inner speaking makes use of all three kinds of representations. However, does IS consist in all of them? If IS is characterized in product terms, it seems that IS has to be strings of phonological *acoustic* representations. There are two reasons to support this claim. Firstly, if the format has to be introspectable/perceptual, it seems that only acoustic representations can do the trick, given that neither articulatory nor phonemic representations are introspectable according to his account (see above). So, following Jackendoff, Prinz states that speech sounds, where he includes silent speech, “are experienced at a level that lies above the buzzing confusion of unfiltered sound waves but below the level of phoneme categories” (Prinz, 2012, p. 69).

Secondly, some authors believe that IS as a product makes thoughts conscious because IS is a prediction issued on the basis of an afterward aborted motor action (see Carruthers, 2011; Pickering and Garrod, 2013). Subjects give instructions to produce a certain linguistic item; these instructions are converted into motor commands; and then the command is aborted, but not before an efference copy is sent to the forward models, which issue a prediction about the sensory incoming signal corresponding to the aborted motor command. If this is what IS ultimately consists in, i.e., the prediction of an incoming sensory signal, then, arguably, an instance of IS has to be an acoustic representation, since the prediction represents sounds (not phonemes or articulations).

Be it as it may, we are ready to accept that the association between the strong consciousness and the format theses is more central for the format view than the product thesis, and that any commitments to a certain kind of product typically arise as a consequence of endorsing the two former theses. Indeed, it is only by relaxing these theses that a defender of the format view will be able to deal with some of the challenges for that view that we are going to present.

## PROBLEMS FOR THE FORMAT VIEW

We want to present three general problems we see related to the format view—general in the sense that they stem from endorsing its theses (i) and (ii) (strong consciousness and format). First, it has to deny the phenomenon of “unsymbolized thinking” (UT; Hurlburt and Akhter, 2008). Second, it cannot easily explain how IS makes thought-contents available to consciousness (Jorba and Vicente, 2014). Third, it may have problems in accounting for the variability of uses of IS. In addition to these general problems, we will finally examine a particular rendering of the IS-as-a-product idea, namely, the suggestion that IS is an acoustic representation that predicts an incoming sensory signal—a suggestion that has some problems of its own.

### THE PUZZLE OF UNSYMBOLIZED THINKING

Using the method of Descriptive Experience Sampling, Heavey and Hurlburt (2008) reported that people claimed to experience inner episodes in which they had the feeling of “thinking a particular, definite thought without the awareness of that thought’s being conveyed in words, images, or any other symbols” (p. 802). For instance, someone could report her experience as wondering whether a friend would be driving his car or his truck but with no words carrying this specific content, and no images of the friend, the car or the truck (Hurlburt and Akhter, 2008, p. 1364). According to their results this kind of “unsymbolized thinking” occupies around an average of 22% of our conscious life (Hurlburt and Akhter, 2008; Hurlburt et al., 2013).

Unsymbolized thinking is not an uncontroversial phenomenon. Even though there are other strands of research that point toward a distinctive phenomenology for propositional thought (Siewert, 1998; Pitt, 2004), its characterization is elusive. For instance, Hurlburt and Akhter (2008) portray it mostly in a negative way, holding that “unsymbolized thinking is experienced to be a *thinking*, not a feeling, not an intention, not an intimation, not a kinesthetic event, not a bodily event” (p. 1366). In this paper we do not wish to enter the debate concerning the evidence for UT. Rather, the point we want to make is conditional: *if* UT is a genuine phenomenon to explain, it poses a serious problem for the format view. This view claims that we recruit IS so that we can have conscious thoughts—otherwise, we would not be able to think consciously. But if it is possible to have conscious thoughts without the presence of IS then the format view’s claim is simply false. Indeed, its best strategy is simply to deny this phenomenon. In this vein, Carruthers (2009) argues that UT may be a result of confabulation: people report thinking without words or images, but they may be actually using words and/or images, or they may not be really thinking (e.g., they think that they were thinking about what product to buy, but in fact they were only looking at the different products). Hurlburt et al. (2013), in contrast, suggest that confabulation probably goes the other way around: we engage in more UT than that 22% average, but as we tend to identify thinking with innerly speaking, we tend to report using words when in fact we are not using them.

To repeat, any view that endorses both the strong consciousness and the format theses will hold that, in fact, IS is the form that conscious propositional thinking adopts^7^, so inasmuch as UT is propositional it is simply impossible. However, it is possible to construe weaker versions of the format view in which UT appears as a more tractable phenomenon. In particular, one may drop the *strong* consciousness thesis and hold that IS is not *necessary* to have conscious thoughts. IS would be only a good way, possibly the best, to make thoughts conscious, but there are other ways to do so. Perceptual theories of consciousness (Prinz, 2011) are a good candidate for this weaker version. These theories claim that a thought always needs a certain perceptual format in order to be conscious, and that “even high-level perceptual states and motor commands are inaccessible to consciousness” (Prinz, 2011, p. 174). IS constitutes a variety of such a perceptual format but there could be others. In particular, there could be *non-symbolic* perceptual vehicles, like emotions, or bodily feelings. Following this path, there is a chance to account for UT without denying the phenomenon: an unsymbolized thought would be a thought that is cashed-out in some non-symbolic perceptual format.

There are problems for such an account. A first problem is that it is not clear that it actually fits the characterization of the phenomenon offered by researchers of the phenomenon. Recall that Hurlburt and Akhter (2008) reject that UT is experienced as a feeling, intention, intimation, kinesthetic or bodily event, adding that people “confidently discriminate between experiences that are thoughts (…) and experiences that are feelings (…) or sensory awareness” (p. 1366). This seems to leave very little room to manoeuver for a perceptual account of UT. Now, one may protest that Hurlburt and Akhter’s (2008) positive characterization of the phenomenon is somewhat wanting and that there is perhaps a different kind of perception behind it. So let us focus on a second problem that seems to be more pressing for the perceptual account, namely, the problem of accounting for the specific semantic content of the unsymbolized thoughts that subjects report.

If UT is a genuine phenomenon the only positive characterization we have is that subjects claim to be experiencing definite thoughts^8^. So any account of the phenomenon will have to respect this characterization. Consider the unsymbolized wondering whether a friend would be driving his car or his truck. What sort of perceptual experiences could carry that content? If the subject were engaged in an experience of IS the answer would be straightforward: it is the content of a mental sentence. But non-symbolic perceptual experiences, such as certain feelings associated to your friend and his truck, appear as unsuitable for that task. Certainly, in Prinz’s view (e.g., in his theory of emotions, Prinz, 2004) feelings can have intentional contents, but they do not seem to be so nuanced to include the specific content of a thought such as the subject’s wondering. Prinz’s suggestion of treating propositional attitudes in terms alike to emotions (Prinz, 2011) can help with respect to the “attitude” part, i.e., it might be the case that what distinguishes “wondering whether *p*” from “doubting that *p*” is a certain emotion-like feeling that accompanies the thought. Yet this feeling does *not* account for the experience of the content *p*, so something else must back the latter experience. Given the problems of attaching specific propositional contents to visual or other non-verbal sensory elements (more on this in the next section), Prinz does not seem to have other resources than imaged sentences. Therefore UT appears for him as unlikely as for other defenders of the format view.

Perhaps a way out of this problem is to claim that the non-symbolic perceptual format is recruited, but not to broadcast thought-contents, but to *prompt* them. That is, perceptual experiences would not be used as *vehicles* of the content but only as means to focus our attention or to keep track of our thought processes. Conscious thinking may thus be unsymbolic in Hurlburt’s sense, even though many times unsymbolic conscious thinking uses perceptual scaffolding. Yet, this alternative view seems full of problems.

The format view provides an account of how IS is generated, and tries to explain how IS makes conscious thinking possible. Yet it has no explanation about conscious thinking which is not supported by IS—the prompting model appears as an *ad hoc* addition to it. If we take Carruthers’s model as a paradigm of the format view (see below), it is clear that the model is not made to explain that IS *prompts* conscious thoughts, but to explain that IS *vehicles* conscious thoughts. Producing a string of phonological representations with contents attached *is* having a thought, according to the model, whereas the prompting model would say that producing a perceptual surrogate—verbal or otherwise—just *facilitates* having a thought in consciousness, the relation between the prompt and the content being arbitrary.

Finally, the format view also seems to account for the sense of agency related to mental phenomena inasmuch as it construes them as motor phenomena. For instance, in Carruthers’s model agent awareness is explained on the basis of the production of imagery that engages the forward model system. The details of how the sense of agency emerges are not clear^9^, yet it seems that the prompting model cannot explain why prompted thinking would feel as our own thinking. The only thing that one would feel as his own would be the prompt.

### HOW THOUGHT-CONTENTS ARE AVAILABLE TO CONSCIOUSNESS

Even if one disputes the evidence for UT, the format view still has the problem of explaining how thought-contents are available to consciousness (see Jorba and Vicente, 2014, for extended discussion). Any account of conscious thinking has to explain how thought-contents become access-conscious^10^. Defenders of the format view hold that by producing strings of phonological representations we bring thought-contents to consciousness. Yet, it is not explained how this is done. It seems that by speaking to ourselves we become conscious of the phonological structure of our IS. How does this kind of consciousness explain consciousness of meanings, or contents? Remember that, on some accounts, like Jackendoff’s, conceptual structures and therefore meanings and propositional contents, are necessarily unconscious. The question then is: how do these structures or representations become conscious, at least, access-conscious, by virtue of making phonological structures conscious?

Clark (1998), as well as Bermúdez (2003) and Jackendoff (1996, 2012) propose that phonological representations convert propositional contents into objects that become present to the mind’s eye. However, it seems that converting a propositional content into an object one can “look at” only enables subjects to know what they are thinking, not to think those thoughts consciously. Instead of making them aware of a certain propositional content *p*, and so to consciously believe or judge that *p*, this mechanism makes them aware that *they are thinking* that propositional content, i.e., that they are believing or judging that *p*. Objectifying seems to give the subject metarepresentation, but not ground-level conscious thinking.

Let us clarify this point in terms of Clark’s position. Clark (1998) presents his view as a development of Vygotsky’s ideas about IS Vygotsky (1987). However, the role he envisions for IS is very different from Vygotsky’s emphasis on the role of IS in self-regulation and executive on-line control, as well as in planning more or less immediate actions—that is, not planning a summer trip, but planning how to solve the Tower of Hanoi task. Vygotskyans typically hold that IS helps us focus our attention on what we are doing, whereas Clark et al. hold that it makes possible for us to focus on what we are thinking. Vygotskyans point out that IS is involved in, inter alia, executing an action step by step. This means that IS enables us to do whatever we are doing in a conscious mode. We monitor our behavior by consciously thinking “this goes here,” “this goes there,” “if this goes here, then that goes there,” etc. In contrast, Clark’s model is a model not of behavior control or monitoring, but apparently of metacognition, i.e., of knowing what we think. We believe there is a difference between saying that IS helps us to have conscious thoughts, which are used to monitor and control our behavior, and holding that IS makes us aware of what we are thinking, so that we are able to think about our thinking.

Perhaps Clark, Jackendoff and Bermúdez do not intend their account to have the narrow scope we are ascribing to it^11^. However, the model they propose seems to only be able to explain how IS gives us knowledge of what and how we think. Let’s say that by using sentences of our language, we are able to have some kind of object before our minds. What do we gain with that? Presumably, we only gain knowledge about what we are thinking. We “see” the sentence, get its meaning, and reach the conclusion “ok, I’m thinking that *p*.” This knowledge about what and how we are thinking may be very useful, of course, but we would say that this is only a use of IS, among many others^12^. The account, in any case, does not explain how thought-contents are made access-conscious.

In this respect, Carruther’s (2011, 2014) idea that thought-contents are bound into strings of phonological representations and broadcast along with them fares much better. For according to this idea, thought-contents as such make it into access-consciousness by being bound to formats which are both phenomenal and access-conscious: “there is every reason to think that conceptual information that is activated by interactions between mid-level areas and the association areas (…) gets bound into the content of attended perceptual states and is broadcast along with the latter. Hence we don’t just see a spherical object moving along a surface, but a tomato rolling toward the edge of the counter top; and we don’t just hear a sequence of phonemes when someone speaks, but we hear what they are saying; and so on” (Carruthers, 2014, p. 148).

What is not clear in this view is how the binding process takes place, especially given that, according to Carruthers, what we do in order to extract the meaning of an IS episode is to interpret an already conscious phonological representation by means of the usual comprehension mechanisms. According to Langland-Hassan (2014), however, the only content that can be bound into an episode of IS is of the kind: the semantic meaning of this episode of IS is such and so. That is, the content bound into the string of IS would not be about the world, as it should be, but about the very string^13^. The reason is, basically, that phonological representations represent acoustic properties, while semantic representations represent the world. Langland-Hassan argues that there is no way to fit these different kinds of representations into a single item.

There are perhaps reasons to resist this idea. If one regards representational content as the information that a representation conveys, it is clear that a representational instance can convey different kinds of information. A phonological representation may represent sounds but it is by means of this acoustic information that it also represents certain semantic information. That is, in a nutshell, Prinz’s position (Prinz, 2011, 2012). Prinz argues that consciousness requires attention to sensory representations. These representations are “images generated from stored concepts [that] inherit semantic properties from those concepts” (Prinz, 2011, p. 182). IS constitutes a particularly important kind of images, i.e., linguistic images, which carry information *both* about acoustic properties and semantic content. In this respect, Prinz’s theory seems to eschew Langland-Hassan’s criticism: causal-informational chains are responsible of keeping the different sorts of information attached to the same sensory representation, so the binding problem may not arise.

However, Langland-Hassan’s analysis also raises another concern: those different contents have different functional or inferential roles to play. Acoustic information will play a role in inferences having to do with the representation’s sound, while semantic information will be routinely exploited for reasoning processes having to do with what those words mean. Those inferential roles cannot simply be mixed together. Again, Prinz’s view may have a way out of this difficulty: those contents are not attended at the same time. To have conscious thoughts, a subject must have a certain sensory representation in mind *and* attend to it, but nothing precludes that at some times she attends to its sensory properties, and others to its semantic content. So thoughts are available to consciousness simply by attending to the sensory elements related to the semantic representation proper.

We think there is a problem in this position. Compare the case in which a subject is attending to the representation’s sensory information with the case in which she is attending to its semantic information. What is the phenomenological difference between both cases in the subject’s mind? According to Prinz’s perceptual consciousness account, there must be some sensory difference between them, e.g., an accompanying sensory representation. So if the subject is thinking about the representation’s acoustic information some acoustic-related representation will be present; if she is thinking about its semantic information, some semantic-related representation will be present.

This account paves the way to an infinite regress. Notice that accompanying representations have to be sensory representations themselves, and the same sort of question can be raised with respect to them: does the subject attend to its sensory or to its semantic information? To distinguish between both cases one must appeal to further distinct accompanying representations, which are sensory representations themselves and which raise the same kind of issue. To put the problem in different terms: if you have a theory in which for a thought to be conscious it must be cashed out in a certain format, then you introduce a gap between the thought’s content and the content of the format itself. What makes the thought conscious cannot be simply the format because there is always the question of how that particular format makes that particular thought conscious.

### THE VARIOUS FUNCTIONS OF INNER SPEECH

The final problem for the format view we want to mention is that it is not clear how it can account for the variability of uses and of kinds of IS. We use IS in most of the kind of situations where we may use outer, or overt, speech (OS). For instance, IS is used for motivating, encouraging, entertaining, expressing the speaker’s emotions or feels, guiding behavior, etc. The main difference is simply that OS can be addressed to someone else whereas IS has to be addressed to oneself. So among the functions of OS that we probably would not find in normal IS we can count those actions that conceptually require somebody else, like promising and threatening, perhaps—yet IS can include comparable functions, such as warnings. At any rate, this is just a reflection of how the things one can do with language depend on the audience one is addressing but this reveals no important, or deep, functional difference between outer and IS.

When it comes to explaining the plurality of functions of IS, the format view may have a problem. The format view is not committed to claiming that we only use IS for having conscious thoughts. However, apparently, it does propose a story about why IS is recruited and thus seems to commit to a certain idea about the *proper function* of IS: the proper function of IS would be to make conscious thinking possible, while uses of IS not related to conscious thinking would be derivative. Yet it is difficult to see how such derivation would proceed. For instance, if one considers the case of OS, one cannot find an analogous fundamental function. One might appeal to the notion of “communication,” arguing that it is akin to the very general function of “focusing someone else’s attention on something,” or “making someone conscious of something.” Yet this is at most a loose way of speaking.

Let us flesh out a general motivation that lends support to the thesis that IS may have a proper, constitutive, function. There is this old conundrum about why someone ought to talk to herself, when she knows in advance what she is going to say. In other words, if one thinks that the semantic content is “already there” before the words are actually uttered, one should not bother to put it in words for oneself. In other words, IS cannot have a communicative function because communication presupposes an informational mismatch between the speaker and the listener, and this mismatch does not exist when both roles concur in the same person. Second, it is not clear that some uses of IS count as communication. For instance, it does not seem to be necessary to characterize self-motivation, or even self-evaluation or self-awareness (Morin, 2011), in terms of communication. It is weird to say that when you motivate yourself with words you are engaging in some act of communication with yourself. If IS does not have a communicative function it must have a function of its own. Which one? A promising response seems to be that IS has a function related to conscious thinking.

Even though this is an alluring motivation, we think it has a basic flaw: it seems to assume that the function of outer speech is *merely* communicative. However, this is not the case. OS can play the same cognitive roles as IS, including the alleged roles related to consciousness. When the mother, helping her daughter to solve a jigsaw puzzle, tells her “this here… that there,” etc., she is directing her attention to the items and the places, i.e., she is regulating her behavior by talking, just as we are supposed to be doing when we use IS. In principle, anything that we tell in IS could be told in OS, and for exactly the same purposes. So if IS had the function of making thought-contents conscious, it would certainly not be its proper function but a function of speech in general (e.g., in the case considered, we can say that the mother is making her daughter conscious of where the different pieces go, so that the daughter consciously judges that this piece goes here, etc., thus gaining control over the resolution of the puzzle). IS would not have the communicative function of OS but IS’s functions could still be considered as a subset of OS’s.

However, this “proper function” commitment may be not essential to the view. It is relatively easy to read authors as endorsing claims about the proper functions of IS—many statements take the form of “we use IS for x,” where x is substituted by conscious thinking, system-2 thinking (Frankish, 2010), self-regulation, executive control, or whatever. Yet, it may be non-charitable to read these claims as expressing strong views about proper functions. A more liberal reading is to think that each author has focused on a use of IS and has simply apparently left the rest in the background. We think it is methodologically advisable to start by first detailing the different uses of IS, the different situations where we use it, as well as the different kinds of IS that there may be, but this is a different issue (for examples of this kind of approach, see Morin et al., 2011; Hurlburt et al., 2013). The point now is that defenders of the format view may drop a strong commitment to a proper function of IS and accept a plurality of uses.

However, even if the “proper function” commitment is abandoned, we think that when it comes to account for the uses of IS the format view typically has the order of explanation backward. The story assumes that IS couches thoughts in a certain format, and that, by doing so, those thoughts can be put to new, different uses. Yet the functional order is just the opposite: thoughts are formed and recruited to be put to different uses and, in doing so, they can appear in a certain format. Consider the example of an athlete telling herself motivating words (Hatzigeorgiadis et al., 2011). The athlete does not first form the mental sentence “you can do it” and then use this sentence to motivate herself. Rather, the athlete is engaged in the activity of motivating herself and, in doing so, her motivating thoughts can reach the point in which she hears herself telling encouraging words silently (or even aloud sometimes). Or consider the case of someone deciding to put more money in the parking meter and telling himself “One more quarter? Mmm… Can be back in one hour. Better a coffee.” The subject is making a decision by means of certain conceptual activity. Some of the elements of this activity—typically the most salient and relevant ones—can emerge to consciousness under verbal control, where they can be put to further uses and lead to new cycles of mental activity. These two examples are cases in which the linguistic production system may be recruited spontaneously so that, so to speak, “words come to our mind” but, of course, we can also *bring* words to our minds by engaging explicitly in linguistic activity. The student preparing a talk may revise innerly some of the sentences she intends to utter, so as to change a few words, decide where to put the emphasis, and the like. Again, the way of describing this is not that she is putting her thoughts in verbal format and then examining them. Rather, she is already engaged in the activity of examining her own thoughts on the matter she wishes to talk about and uses her verbal systems so as to do this in a more precise manner.

On the other hand, endorsement of the format view involves that, even if one abandons the idea of a proper function, one still holds the claim that recruiting a format plays a necessary role in the plurality of functions. Yet some of those functions cast doubts concerning the claim that the format is necessary—let alone the linguistic format. Think again about IS and motivation, which is amply discussed in the psychology of sports literature (Hatzigeorgiadis et al., 2011). An athlete does not need any kind of particular format to motivate herself: she may tell herself “give it all!!,” but she could just as well fix her sight at the finish line and see how close it is, feel how fast her legs are moving, or whatever. She needs perceptual or proprioceptive stimuli, but these do not have to be self-produced (i.e., they do not have to be the result of imagery or IS production).

Finally, the idea that in IS we always recruit a format for a purpose is also open to doubt. There seem to be cases where the only thing we do with IS is add a clearly unnecessary expressive commentary to something that we have done (Hurlburt et al., 2013), like the ‘a-ha’s, or ‘great!’s we tell ourselves after, for instance, having thought hard about something. Would we say that, in these cases, we are recruiting a format with some purpose? Arguably, we would not put it in that way. Moreover, we would probably say that we are using IS with no purpose at all—at least no purpose related to the cognitive activity in question. Yet, non-purposive IS seems to be a problem for the format view however weakly it is construed, for the format view wants that phonological representations are used to perform cognitive functions.

### IS INNER SPEECH A PREDICTION?

In this last section about the problems of the format view we want to consider briefly the particular proposal about IS we have mentioned above, namely, that it is a prediction about the linguistic sounds that one would hear if a certain linguistic action had not been aborted. This proposal has some independent appeal, as it construes IS as a species of motor imagery (Carruthers, 2011, 2014). Current theories of motor imagery (Jeannerod, 2006) hold that motor imagery results from aborting the execution of motor commands, and from generating a prediction about sensory and proprioceptive incoming signals. It is appealing, we think, to embed IS in a larger theory about imagery production.

However, the proposal that an episode of IS is a prediction about linguistic sounds does have some problems. One first problem is that it cannot accommodate the intuitive idea that IS is typically experienced as *meaningful*, e.g., when one is engaged in conscious reasoning. This is in contrast with meaning-ignoring instances of IS (e.g., when one repeats some linguistic items mentally so as to memorize them—we will call these cases “meaningless” for short). We would say that when we talk about IS in contexts like the present one, we are only talking about meaningful IS. However, the way the format view prefers to individuate IS does not need semantics, meaning or content—or if it has a role for semantics, it is a secondary one, ancillary to the format’s properties. So both meaningful and meaningless instances of a string of phonological representation could count as the same type of IS.

The proposal also seems to have problems to deal with data which apparently show that IS may contain errors which are recognized as such (Oppenheim, 2013), because, prima facie, a prediction issued on the basis of an efference copy is not monitored; rather, its proper function is monitoring production. A related, and complicated problem, is that the proposal excludes the currently widely accepted idea that passivity phenomena in cognition (auditory verbal hallucinations (AVHs) and thought insertion) may derive from a misattribution of IS (e.g., Ford and Mathalon, 2004; McCarthy-Jones, 2012; see also Langland-Hassan, 2008, for a revised version in terms of a filtering/attenuation deficit)^14^. This latter idea seems to require that IS is an *incoming signal* against which a prediction is compared, rather than this very prediction. That is, misattribution (as error checking) is only possible when there is comparison, which in turn requires a prediction *and* an incoming sensory signal. If the only product we get from inner speaking is a sensory/acoustic prediction, then it is mysterious how we could self- or other-attribute it (see, however Vicente, 2014 for development and criticism of the idea that IS is an incoming sensory signal). It seems that both error checking and misattribution require that IS is *not* a prediction about linguistic sounds issued by the forward models.

## THE ACTIVITY VIEW OF INNER SPEECH

The view we want to argue for stresses the *activity* of innerly speaking, instead of the format of IS. This view is not without precedent. For instance, the emphasis on activity is a key ingredient in the Soviet school to which Vygotsky belongs (Kozulin, 1986; Guerrero, 2005) and many contemporary Vygotskyans understand language as activity-based (Carpendale et al., 2009) and IS as an internalization of this activity. Other recent approaches that characterize IS as preserving some feature of linguistic activity—and not merely linguistic format—include Fernyhough (2009), who conceives of language as inherently dialogical, or Hurlburt et al. (2013), who commend the use of inner *speaking* to avoid regarding IS as mere representational product.

In relation to the format view we depict in this paper, our idea of an activity view of IS rejects both the format and the strong consciousness theses associated to the former. With respect to the format thesis, it claims that in IS we do not recruit a format, be it perceptual, predicative, or whatever. At most, we could say that we recruit a linguistic activity, though we think using the notion of recruitment mischaracterizes the view: we do not properly recruit the activity of speaking; we just speak, although innerly. With respect to the consciousness thesis, the view denies that IS is necessary for thinking consciously, or that IS is *for* thinking consciously (i.e., that its proper function is conscious thinking). Rather, the activity view adopts a pluralistic stand: IS has almost as many functions, or uses, as we can discover in OS, none of which should be singled out as its proper function.^15^.

If we observe our own IS we will see that, in effect, IS is put to use in many different circumstances: self-expression, motivation, evaluation, attention-focusing, self-entertainment, fixing information in memory, preparing linguistic actions, commenting on what we have done, accompanying our thoughts, etc.^16^. There seems to be no deep difference between reasons why we talk to ourselves and reasons why we talk to someone else: we talk to express ourselves, to motivate others, to evaluate events or subjects, to help people to find places, to regulate their behavior, etc. Moreover, there seems to be no deep difference between the way we talk to ourselves and the way we talk to someone else. For instance, if we want to motivate our favorite athlete, we may tell her “come on!,” “you’re the best!,” that is, the kinds of things she may be telling herself. If we want to help someone to get to a certain destination, we may use a map and tell him “you go here, then there. Go straight this way, turn here,” etc. That is, we insert linguistic fragments within the background provided by the map, which is what we do when we mix mental maps and IS in orientation.

There are also parallels between the cases in which IS and OS appear in longer, more elaborate linguistic constructions vs. those in which they appear condensed or fragmentary. For instance, when we talk about ourselves, or about a certain person or event that concerns us, we typically use full sentences, and elaborate a narrative, just as we do when we get introspective about ourselves, other people, or certain events. On the other hand, our speech appears as condensed or fragmentary if we are regulating someone else’s behavior on-line: the adult that helps his kid to complete a jigsaw puzzle, tells him “this piece here. Square there? Sure? Where is a triangle missing? No. Yes,” etc. As has been long highlighted by Vygotskyans, IS, when put to this kind of use, is equally typically condensed^17^. This suggests that using IS is, basically, innerly *speaking* (see also Hurlburt et al., 2013).

The activity view we propose is in clear contrast with the strongest versions of the format view, i.e., those which hold that IS is for conscious thinking, and that IS is necessary for conscious thinking because we need a certain format to get thought consciousness. However, in the discussion of the format view we have considered weaker versions of it. A weak version of the format view, for instance, could simply claim that we produce phonological representations to better do a variety of things, from conscious thinking to motivation. The activity view and this weak version of the format view do not look that different in principle.

However, there are reasons to prefer to categorize IS as an activity *tout court* rather than in terms of a format. First, labeling IS as an activity fits better the natural description of IS as speaking, and not as producing phonological representations (even if phonological representations are produced). Second, the notion of activity underscores the functional continuity between outer and IS in a more natural way than the format view. As we explained, the format view typically begins by focusing on a function that is putatively exclusive to IS, i.e., thought consciousness. The consequence is that it tells apart outer and IS—the former is an instrument of communication, the latter of cognition. Even if one relaxes the account to make it sensitive to the plurality of uses of IS, it tends to consider these uses as solutions to particular cognitive demands. The activity view, in contrast, regards them as predictable effects of internalizing OS and its different functions.

Be it as it may, the view we want to propose deserves the label “activity view” on further grounds, which mark a stronger contrast with the format approach. We claim that IS, as speech in general, is characterized as a *kind of action*, namely, an action that consists in expressing thoughts. In philosophical parlance, this means that IS is *individuated* in terms of the action it is, i.e., that it is distinguished from other mental phenomena attending to what the person (or the person’s mind) is doing. This excludes that IS should be individuated in terms of its product qualities, e.g., its properties as a string of phonological representations.

The question of how to individuate IS is not a mere metaphysical point but has important methodological consequences about how one should approach its study or what sorts of mental mechanisms are relevant for it. For instance, by laying the focus on the action of speaking, it is quite natural to try to understand IS in terms of all the representations that are mobilized in speech, i.e., semantic, syntactic, maybe articulatory, etc. As we argued in Section “How Thought-Contents are Available to Consciousness,” in the format view the semantic properties of an instance of IS appear as something that one has to bind to it—not as something inherently constitutive of it—raising concerns about how the binding takes place. In contrast, for the activity view the act of innerly speaking begins with a prior intention to express a certain thought that can get more and more specific, until it reaches the level of motor commands. The representations involved in the activity—from conceptual to phonological—form an integrated system, and the ultimate format’s properties have no privileged role in accounting for the phenomenon and its functions.

## ADVANTAGES OF THE ACTIVITY VIEW

We hold that the activity view has several advantages over the format view. In this section we will develop a particular proposal about how the activity view can explain certain phenomena. The activity view, as we have presented it, is rather liberal in its commitments. Thus, it is compatible with what we have said so far to hold that we do not have to bind thought-contents to phonological representations: it can be said that we interpret our IS just as we interpret OS, i.e., by means of the linguistic-plus-pragmatic system. It is also compatible with the view to have it that, although we sometimes use IS in certain activities where conscious thought is involved, conscious thinking is possible without IS. That is, the spirit of the activity view is consistent with a general model of conscious thinking which has it that conscious thinking is typically unsymbolized: sometimes we speak to ourselves as an aid—but in that case we cannot be said to be thinking in IS, and sometimes we engage in conscious thinking directly (for a sketch of this view, see Jorba and Vicente, 2014).

Here we will pursue a different view according to which predictions issued on the basis of high level intentions play a prominent role both in binding contents into phonological representations (or in making IS meaningful) and in explaining UT. On the one hand, we regard this proposal worth exploring because it seems to be able to unify apparently different phenomena. On the other, it is the only proposal that we can think of right now which could explain the nature of UT and the sense of agency attached to it. In all, we think it has more explanatory power than the view we have just mentioned.

### INNER SPEECH AS MEANINGFUL

As we said above, there is a distinction between meaningful IS (involved in the panoply of functions we talked about in the previous section) and meaningless IS (which we use, for instance, in order to simply retain uninterpreted items). If one regards IS as the strings of phonological representations generated by linguistic productions systems, the consequence is that IS is not meaningful *per se*. In other words, the distinction between meaningful and meaningless instances of IS has to be accounted for in some additional mechanism, for instance, an attentional mechanism that puts the focus either on the semantic or the phonetic information of the representation—which, as we argued, poses an explanatory problem. In contrast, the activity view regards meaningful and meaningless IS as different kinds of actions. It is not the case that a subject produces a certain phonological representation and then puts it to different uses, or under different attentional processes. Rather, the very production of the phonological representation starts with different intentions that mobilize different sets of representations, e.g., in the case of meaningless IS semantic representations are simply not mobilized to begin with. In concordance with this approach, we think that the notion of inner *speech* proper corresponds only to its meaningful instances^18^.

Another related advantage is that, by insisting on the idea that IS is inherently meaningful, the activity view easily avoids one aspect of the binding problem we mentioned in Section “How Thought-Contents are Available to Consciousness.” As we pointed out above, it is not easy to see how something that represents sounds may also (semantically) represent the world. So if we individuate IS in terms of format properties, we have to explain how content gets bound to it. In contrast, according to the view we are proposing, IS proper is meaningful, and content is an integral part of IS episodes—it does not appear as something “external” that one somehow attaches to represented sounds. Moreover, we are in a position to claim that the content of an IS episode is not the content that phonological representations could eventually encode, but the content that the subject intends to express. In other words, the activity view agrees that in IS the content eventually adopts a certain format, but the specific properties of the format are secondary to explain the phenomenon.

This issue turns out to be particularly important when we consider condensed or fragmentary IS: a linguistic fragment (say, “the ball!”) can be used to express many different thoughts (that I lost the ball, that you lost the ball, that we left the ball at home…). Most utterances, if not all, can express different thoughts, depending on the circumstances, but fragments are especially ambiguous (Vicente and Martínez-Manrique, 2005, 2008; Martínez-Manrique and Vicente, 2010). Now, how can we say that the string of phonological representations that constitute “the ball!” means, e.g., that we left the ball at home? It only conveys this specific content if we take into account not the representations themselves but the intentions of the speaker. It seems to us that this sort of response is not so easily available for format views. In particular, the position we attributed to Prinz above may have trouble in explaining how the intended content (i.e., the content subjects want their words to have in a particular occasion) gets bound into the phonological output.

### BINDING AND THOUGHT CONSCIOUSNESS

There is another aspect to the binding question, however. In fact, it is this other aspect that occupies Carruthers (see How Thought-Contents are Available to Consciousness). Recall that Carruthers resorts to binding in order to explain how thought-contents become access conscious. His view is that thought-contents can be bound into phonological representations and be broadcast together with them. Carruthers, thus, is not so much concerned with how phonological representations have meaning as with how this meaning is broadcast and made available to higher-level cognition. That is, Carruthers’s binding account is a response to this latter issue. The question, then, is: can the activity view do better than Carruthers’s version of the format view in this respect? We want to argue that it can.

In motor imagery, as well as in motor acts, the brain issues efference copies and predictions, which are used to monitor and eventually correct actions on-line, as well as to confirm authorship (Jeannerod, 2006). It is not yet clear how the sense of agency arises (see The Puzzle of Unsymbolized Thinking), but it seems likely that it is linked to the good functioning of the forward-models system of efference copies and predictions. Now, less is known not only about so-called mental actions, but also about how the system handles higher-level intentions. However, one can claim that the system does not only receive efference copies from motor commands and issue predictions about incoming sensory signals; it also has to receive efference copies from higher-order intentions and to make predictions on that basis (see Pacherie, 2008).

The architecture for the comparator system proposed by Pacherie (2008) involves a hierarchy of intentions and predictions. This allows her not only to explain how it is possible to monitor the execution of higher-level intentions, but also to provide an account of the different components of the sense of authorship. Pacherie distinguishes three levels of intentions: distal, proximal, and motor intentions (motor commands). Distal intentions are about the goal of the action; proximal intentions are about the here-and-now execution of the distal intention; and motor intentions are about the movements of the body that will eventually realize the proximal intention. As she says, each kind of intention deals with a particular type of representation: “The contents represented at the level of D-intentions as well as the format in which these contents are represented and the computational processes that operate on them are obviously rather different from the contents, representational formats and computational processes operating at the level of M-intentions” (Pacherie, 2008, p. 192). According to her, distal (D) intentions work with propositional/conceptual representations; proximal (P) intentions with a mixture of conceptual and perceptual representations; and motor (M) intentions with analog-format representations.

We do not want commit to the specifics of Pacherie’s proposal, but we think that her points about (i) the different levels at which the comparator system works, and (ii) the different kinds of representations accessed at each level, are both sensible points. It is at least sensible to think that a monitoring system such as the comparator system has to allow for multiple levels of control. Subjects have to track not only how motor commands are executed, but also whether the intentions that triggered such motor commands are being realized as expected and predicted. Now, we can apply this kind of model to speech generation in general, where the action of speaking begins with an intention (which would be the D-intention) to express a certain thought and culminates with the production of a string of sounds. Speech-related intentions at the different levels generate predictions via the forward model system, which are used to check whether the speech action is being properly realized.

A hypothesis suggests itself at this point: the predictions linked to prior intentions may be made conscious in the same way that we can presumably make conscious the predictions linked to motor commands. Unless we accept a ban on making non-sensory predictions conscious, there is apparently no reason to suppose that we could not make this kind of prediction conscious. Carruthers holds that predictions (sensory predictions, in his case) are made conscious by focusing our attention on them. In general, Carruthers (like Prinz, 2012) believes that consciousness requires attention. There are other hypotheses, though. Jeannerod (1995), for instance, claimed that predictions are conscious just by being predictions of aborted actions, i.e., if an action is aborted after the prediction is issued, the prediction will make into consciousness. His argument is that, when a motor command is aborted, “the motor memories are not or incompletely erased, and the representational levels are kept activated: this persisting activation would thus be the substrate for (conscious) motor images” (Jeannerod, 1995, p. 1429). In any case, our suggestion is that the mechanism that makes sensory predictions conscious may also work for non-sensory predictions.

If this were true, then we may claim that what is made conscious in IS is not just phonological representations, but also their meaning. The prior intention in an act of speaking consists in intending to express a certain thought-content. The prediction corresponding to this kind of intention is the semantic content of the utterance: what we predict, and what we monitor, is that a certain thought-content is expressed. If we were able to broadcast this prediction along with the sensory prediction (i.e., the phonological representations), there would be no need for a further binding of contents into sensory predictions. This seems to be allowed by a theory such as that sketched by Jeannerod (1995), where predictions are conscious by default, but it is more problematic if we follow Carruthers’s idea that consciousness requires attention. The trouble in this case is that to be conscious of meaningful IS we would need to attend to two kinds of predictions simultaneously: a prediction about a content, and a prediction about some sounds. In our discussion of Prinz’s view in Section “How Thought-Contents are Available to Consciousness,” we argued that this kind of scenario is not feasible. Yet, we suggest that it is possible to direct our attention not to this or to that particular prediction, but to the outputs of the forward systems (i.e., what the forward systems deliver) considered as a whole. After all, the predictions corresponding to the different layers of intentions are simultaneously active, given that all of them are used in monitoring both the eventual incoming signal and the predictions lower in the hierarchy. This means that the outputs of the forward systems—the cascade of predictions of different levels—form a close network or integrated whole^19^.

### THE RELATION BETWEEN INNER SPEECH AND UNSYMBOLIZED THINKING

The explanation we just have just outlined has the interesting consequence of allowing us to think about UT in terms of IS without collapsing the former into the latter. In contrast with the format view, the activity view can easily accommodate UT, as this view does not require that a certain format be used for thinking consciously (see Jorba and Vicente, 2014). This is another advantage of the activity view, namely, that by seeing IS as, simply, internal speech, it is not committed to any claim concerning whether or not conscious thinking and phenomenology are possible without a perceptual/sensory medium. However, here we want to move a step further and propose a speculative, though we think plausible, explanation of what UT may be which makes it continuous with IS and begins to account for why we feel authorship with respect to our conscious, but unsymbolized, thoughts (like the judgment that my friend is driving a car).

We just said that it is reasonable to think that the forward system also generates predictions about the likely contents of an utterance. Maybe, we have speculated, this kind of prediction can also be made conscious. Suppose now that we abort a speech action before orders go downstream to motor commands. Then we might get a broadcast prediction about the content of the utterance, which would be experienced as a thought (since it is composed by conceptual/meaning representations). Moreover, there is some chance that it would be experienced as an action because it engages the forward system. At least, minimally, an unsymbolized thought under this construal would feel as initiated (will have the feeling of initiation), as there is an intention in its etiology—which, plausibly, would not be there if we construe UT as simply thoughts (apparently, a thought is not produced by the intention to have it). But it is possible to hold that it would be felt also as authored. As we explained in Section “Is Inner Speech a Prediction?”, it is typically said that the sense of agency requires successful comparisons, usually between sensory predictions and sensory signals. But perhaps the comparison between a goal state and a high-level prediction is enough to generate a feeling of agency. Even if not much is known about how the sense of agency is generated in the mental realm (Frith, 2012), we think the possibility that mental agency is related to comparing high level “products” is worth considering.

If we conceded this view, UT would appear as closely related to IS^20^. We think that this fits nicely the phenomenological characterizations of people reporting UT, in which the subjects have no problems in giving a precise verbal, propositional characterization of what they were thinking yet resist the suggestion that they were experiencing those contents verbally. This easiness of propositional report makes sense if UT is roughly the beginning of a speech act that never became verbally realized. Moreover, the account also advocates a continuity that goes from UT to private speech. Taking into account Vygotsky-inspired approaches, it is not advisable to separate private speech from what we usually call IS, or even from UT, so we see this as a further advantage of our way of looking at IS. The difference between, say, typical IS and muttering, or even private speech, is not a difference in functionality: muttering serves the same general functions as IS (motivation, focusing attention, self-evaluation, etc.). The difference lies in that in typical IS we allegedly produce a prediction about phonological acoustic representations whereas in muttering and in private speech we produce actual sounds. In muttering and private speech, besides, we engage articulation more clearly. In contrast, according to our proposal, in UT we do not even reach the phonological level. Vygotsky claimed that IS is typically condensed with respect to outer speech, and that it is possible for adults to push this condensation to its limit, being able to think “in pure meanings” (see Fernyhough, 2004 for a model of how condensation would proceed). The account here presented would give flesh to this intuition, even though this point of contact with Vygotsky should be regarded as a coincidence (and there are many points of departure from the Vygotskyan tradition: to begin with, UT would not be IS hyper-condensed, but IS aborted before intentions get precise enough). Whether we use one kind of IS, including UT, or the other may depend on stress, the level of attention required, and so on, as Vygostkyans have long claimed^21^.

## CONCLUSION

We have distinguished two general approaches to the phenomenon of IS: the format and the activity view. The format view, as endorsed by authors such as Jackendoff, Prinz, and Bermúdez, among others, holds that in IS we recruit a certain format in order to bring thoughts to consciousness. These authors, as well as others who are not particularly interested in the cognitive functions of IS, think about IS as a product, namely, the strings of phonological representations we seem to experience when we talk to ourselves. We have criticized this position on several grounds: first, it has to deny the possibility of conscious UT; secondly, it does not have a clear account as to how thought-contents make it into access-consciousness; and thirdly, it has too narrow a view about the uses of IS. The format view can be weakened in some dimensions, but some problems remain. UT and the agentive experience attached to it remain unexplained, and the issue of how IS makes thoughts conscious is not improved. On top of these general problems, the hypothesis, endorsed by some authors, that IS-as-a-product is a prediction about sensory stimuli, has problems of its own: it is difficult to explain how we can discover errors in our IS if IS is a prediction, and this construal of IS seems incompatible with the idea that alien voices and/or thought insertion are misattributed IS: misattribution seems to require comparison, and a prediction cannot be compared with itself.

Our general diagnosis about the source of all these problems is that supporters of the format view have a narrow focus on issues such as what is constitutive of IS, what is its main function, or what sort of process may be responsible for its production. We have presented an alternative we have labeled “the activity view,” which takes a more inclusive view on the IS phenomenon. Describing IS as an activity, namely, speaking, amounts to saying that IS is functionally continuous with overt, or outer, speech. We do not recruit a format with some cognitive purpose, but we speak to ourselves in most of the kinds of situations we speak to other people (self-expression, motivation, attention-focusing, behavior-control, having fun, making irrelevant comments…). This description of what we do in IS suggests that we should think about IS not merely as the output of the linguistic production system, but as the whole action of speaking. Speaking is an action that begins with a prior intention to express a certain thought and plausibly finishes with the production of some sounds that have a certain meaning. The typical IS is that kind of action, except that sounds are not produced but simulated. Adopting this more inclusive view on the phenomenon allows us to solve the problems that affect the format view. First of all, thinking about IS as simply speaking does not question the possibility of UT. Secondly, the view has no problem with explaining the conscious access to thought contents. As it allows that we can think consciously without IS, it is compatible with the view that IS is used only as an aid in some circumstances, lending support to other cognitive functions (e.g., focusing attention in a complex task), or prompting further cognitive resources. Finally, the activity view is in good part motivated by the different uses of IS we can discover.

However, in this paper we have explored other explanatory possibilities for the activity view with several objectives in mind: to be able to capture the intuitive idea that IS proper has meaning, to explain how this meaning can be attached to, and made conscious together with, phonological representations, and to address two particularly intriguing problems: the nature of UT and the sense of agency attached to it. The proposal we have presented makes use of the characterization of IS as an action in order to explain the binding problem, the nature of UT, and the sense of agency related to conscious thinking. Concerning the binding problem, we have suggested that individuating IS as an action, which begins with a prior intention to express a certain thought, makes it easier to explain how thought-contents are bound into strings of phonological representations. Prior intentions result in predictions about the content of a thought: if such predictions can be made conscious, we have a conscious thought. If the predictions are made conscious together with predictions about phonological representations we have the typical IS (“the little voice in the head”). If the predictions are made conscious alone because the action is aborted very early on, then we have UT. The feeling of agency in this latter case comes from being a cognitive process that is intended, and, plausibly, monitored.

Finally, although we have not tackled the issue of thought insertion in this paper, we think that this general approach is in an overall better position to explain how thoughts may feel as alien, in a way that is parallel to the detection of errors in IS. Higher-level predictions are used to check the correctness of lower level ones in order to monitor whether higher-level intentions are properly realized. Mismatches may result in misattribution and/or error detection. We regard this idea as material for further research.

### Conflict of Interest Statement

The authors declare that the research was conducted in the absence of any commercial or financial relationships that could be construed as a potential conflict of interest.

## Footnotes

1 Exceptions are Vygotskyans like Fernyhough (2009) and Hurlburt et al. (2013).

2 Along the paper we will introduce a number of weaker versions of the view, which relax one or more theses so as to answer a particular challenge.

3 As the notion of thought has different uses in the literature, let us spell out the properties that matter for this paper:

(i) A thought is a mental state with propositional content.
(ii) It can be individuated from other thoughts in terms of its content.
(iii) It can be unconscious or conscious, so it is possible to have the same thought in both modalities.

A conscious thought, thus, is a conscious mental state with propositional content, for instance, a conscious judgment that *p*. Finally, even if “having a thought” and “thinking a thought” could point toward passive/active occurrences of thought, this is a distinction that we do not discuss in this paper so we will use both expressions interchangeably.

4 See, e.g.: “[Chomsky] has fallen into the trap (…) of believing that inner speech is thought, rather than (as I will argue) the phonological structure corresponding to thought” (Jackendoff, 2007, p. 70), and “conscious thought gets its form (…) from the inner voice, the verbal images of pronunciation” (Jackendoff, 2012, p. 103).

5 We owe this objection to a referee.

6 “Especially important (…) are the auditory images that result from off-line activation of instructions for producing speech, which result in auditory representations of the speech act that would normally result, in so-called ‘inner speech”’ (Carruthers, 2014, p. 149).

7 See, e.g., Bermúdez (2003, (pp. 159–160): “[A]ll the *propositional* thoughts that we consciously introspect (…) take the form of sentences in a public language” (his emphasis).

8 A referee points out that Vygotsky’s distinction between a natural and a cultural line of development is relevant to the question of UT. Those two pathways to thought could result in kinds of thinking with different properties, and UT could occur in both of them, so its analysis would have to take into account the distinction. We agree that this might be the case and insist that a definite characterization of UT is still lacking. In this paper we will limit ourselves to the minimal characterization offered by Hurlburt et al. (2013)—i.e., UT as thought with propositional content and a “proprietary” phenomenological basis—and we sketch a proposal that would link it to the cultural line—see Section “The Relation Between Inner Speech and Unsymbolized Thinking.”

9 As we will see in Section “The Relation Between Inner Speech and Unsymbolized Thinking,” the view that IS is an incoming sensory signal seems to fare better in this respect, for it involves comparisons, which many regard essential to the generation of self-attribution (see Frith, 2012).

10 As it is well known, the distinction between phenomenal and access consciousness was first introduced by Block (1995). Phenomenal consciousness is defined in terms of what-it-is-likeness or experience, and Access consciousness is characterized as information being available to the direct rational control of thought and action.

11 However, see Clark (1998, p. 171): “[P]ublic language (…) is responsible for a complex of rather distinctive features of human thought viz, our ability to display *second order cognitive dynamics*. By second order cognitive dynamics I mean a cluster of powerful capacities involving self-evaluation, self-criticism and finely honed remedial responses (…) This thinking about thinking, is a good candidate for a distinctively human capacity (…) Jackendoff (…) suggests that the mental rehearsal of sentences may be the primary means by which our own thoughts are able to become objects of further attention and reflection.” See also Bermúdez (2003, p. 163): “We think about thoughts through thinking about the sentences through which those thoughts might be expressed.”

12 On the other hand, second-order dynamics and metacognition are probably different phenomena. We can know what we are thinking just by having conscious thoughts: once you think a thought consciously, you also know that you are having that thought. In this respect, thinking is similar to perceiving: when you have a conscious perceptual experience, you thereby also know that you are having that experience. What objectification gives us, we would say, is the ability to reflect about our thinking and to gain control over our higher-level cognitive processes.

13 In philosophical jargon, the content would be token-reflexive.

14 However, Langdon et al. (2009) dispute this claim on the basis of studies with schizophrenic patients. Comparing their AVH and IS, they found no similarities between their phenomenological characteristics—similarities which arguably ought to be present if AVHs derive from IS.

15 The continuity of function between inner and outer speech is a typical assumption in those that understand IS as inheriting the functional roles of the private speech from which it originates (see reviews in Berk, 1992; Winsler, 2009). Relations between inner and outer speech are also currently the focus of attention of empirical research in terms of parallelisms and differences in the linguistic subsystems responsible for their respective processing—e.g., the comprehension and production systems (Vigliocco and Hartsuiker, 2002; Geva et al., 2011). Those topics exceed the purposes of this paper.

16 See Morin et al. (2011) for a study that taps the variety of functions of IS.

17 Vygotsky (1987) and followers have typically been concerned with the use of IS in self-regulation, as they have been particularly concerned with the moment kids start internalizing not just speech but social life in general. Yet, the on-line regulation of behavior is just one function of speech among many others, and it seems that there is no reason why speech should be used only for that purpose when it gets converted into IS.

18 We are aware that one can find a variety of uses for the label “inner speech” in the literature, and we do not mean to legislate the usage of the term. We just want to lay the emphasis on the distinct sort of phenomena that meaningful and meaningless instances are.

19 One might contend that Prinz’s account can resort to this suggestion, i.e., people may attend to both the acoustic and semantic properties of a sensory representation. However, this suggestion does not help Prinz to avoid our criticism of a regress, giving his commitment to accompanying sensory representations.

20 Following what we said in footnote 8, the hypothesis about how UT is generated we are outlining would link it to the cultural line of development by relating it to IS generation. Yet we do not mean to suggest that UT would be impossible if not related to IS. The explanation we put forward about UT could perhaps be extended to the use of any kind of imagery, although it is not clear to us whether purely imagistic thinking can be propositional. Perhaps our account would predict that non-linguistic creatures could not experience UT, as it is usually characterized.

21 Another interesting consequence of this view is related to something we mentioned in Section “Is Inner Speech a Prediction?”. We said that we are sensitive to mistakes in IS (Oppenheim, 2013), which is problematic for the view that IS is a prediction. In our proposal, which contemplates several levels of predictions and monitoring mechanisms, errors could be detected at the level of motor predictions, especially when these, once they are conscious, re-enter the system as inputs. A prediction cannot check itself, but a higher-order prediction can monitor a low-level prediction and detect errors, even more so, we suspect, if the low-level prediction is also treated as an input for the system. We think that the problems we mentioned in that section are motivated by focusing too narrowly on the motor part of the act of speaking.
